# The politics of health systems policies during COVID-19: reflections on experiences from Latin America and the Caribbean

**DOI:** 10.1186/s12939-024-02306-0

**Published:** 2024-11-07

**Authors:** Walter Flores, Alexis Sullivan, Fernando Jerez, Daniela C. Rodríguez

**Affiliations:** 1 School of International Service, American University, Washington DC, USA; 2 Centro de Estudios para Equidad y Gobernanza en Sistemas de Salud, Guatemala City, Guatemala; 3 Johns Hopkins Bloomberg School of Public Health, Baltimore, USA; 4 Independent Consultant, Guatemala City, Guatemala

## Abstract

Politics as the exercise of power always influence public policies–which reflects the multifaceted nature of decision-making–but “using politics” as a motivation for self-serving interests of government leaders and their allies poses problems. This article reviews the impacts of COVID-19 on health systems of the Latin American and Caribbean region from a political lens. We highlight the overriding influence of politics in health policies, weak governance structures that became compromised, exacerbation of corruption, and breakdowns in the communication and trust between governments and their citizens. There are many factors that did not work well-or as expected. For instance, the poor predictive ability of the Global Health Security Index, which showed that pre-pandemic assessments were deeply naive to how health systems evidence and expertise are uniquely vulnerable to politics. We argue that there is an urgent need to rethink health policy and systems frameworks-including metrics-at national and global level. There is also a need for new global health governance arrangements. The expected solidarity and collaboration among countries was trumped by the rich countries practice of gauging essential resources and vaccines and applying health diplomacy to the rest of the world, and the unchecked power of commercial corporations producing essential medical supplies and vaccines.

Politics always influence policies–which reflects the multifaceted nature of decision-making–but “using politics” as a motivation for self-serving interests of government leaders poses problems. Governance, accountability, and citizen participation are critical features of a responsive health system. COVID-19 revealed how tenuous these features are during crisis and highlighted how vulnerable health systems are to power and politicians “using politics”.

Using examples from COVID-19 in the Latin America and the Caribbean (LAC) region, in this Comment we highlight the overriding influence of politics in health policies, weak governance structures that became compromised, exacerbation of corruption, and breakdowns in the communication and trust between governments and their citizens. These features are not unique to LAC but rather emblematic of the broader threat of politics when in support of self-serving interests and their negative effect on health systems and population health. We also see the interconnectedness between social protection measures and downstream effects on health systems and service delivery when faced with uncontrolled disease transmission. These realities should trigger critical reflection on how frameworks and metrics for policy making and emergency preparedness did not meet expectations, and global governance arrangements and mutual development goals have been severely undermined by the lack of international solidarity.

## COVID-19 impact on health systems from political lens

In the 80s and early 90s, LAC was at the forefront of commitments to health and wellbeing, including health system reforms, poverty alleviation efforts, extension of primary health care (PHC), and legal and constitutional commitments to health and health equity, paving the way for universal health coverage (UHC) efforts. These shifts were partly in response to a return to democratic ideals after long periods of dictatorship and unrest. By the late 1990s, neoliberal reforms introduced across the region to decrease costs and increase competition contributed to inequalities in access to and provision of healthcare. By the early 2000s, however, several countries were pursuing counter-reforms to these neoliberal efforts [1].

Notably, health outcome achievements in LAC co-exist with persistent high levels of within-country inequality and marginalization of certain populations, including women, sexual minorities, indigenous and ethnic minorities, and immigrants [2]. The exclusion of indigenous people from policy development and the health system is a critical barrier to achieving UHC in the region [3]. Meanwhile, informal workers, who form a significant proportion of the working population in LAC, are often excluded from health coverage and state welfare [4]. As poor and hard-to-reach populations are excluded from research, it is harder to understand and address health problems that disproportionately affect these populations.

**Fig. 1:**
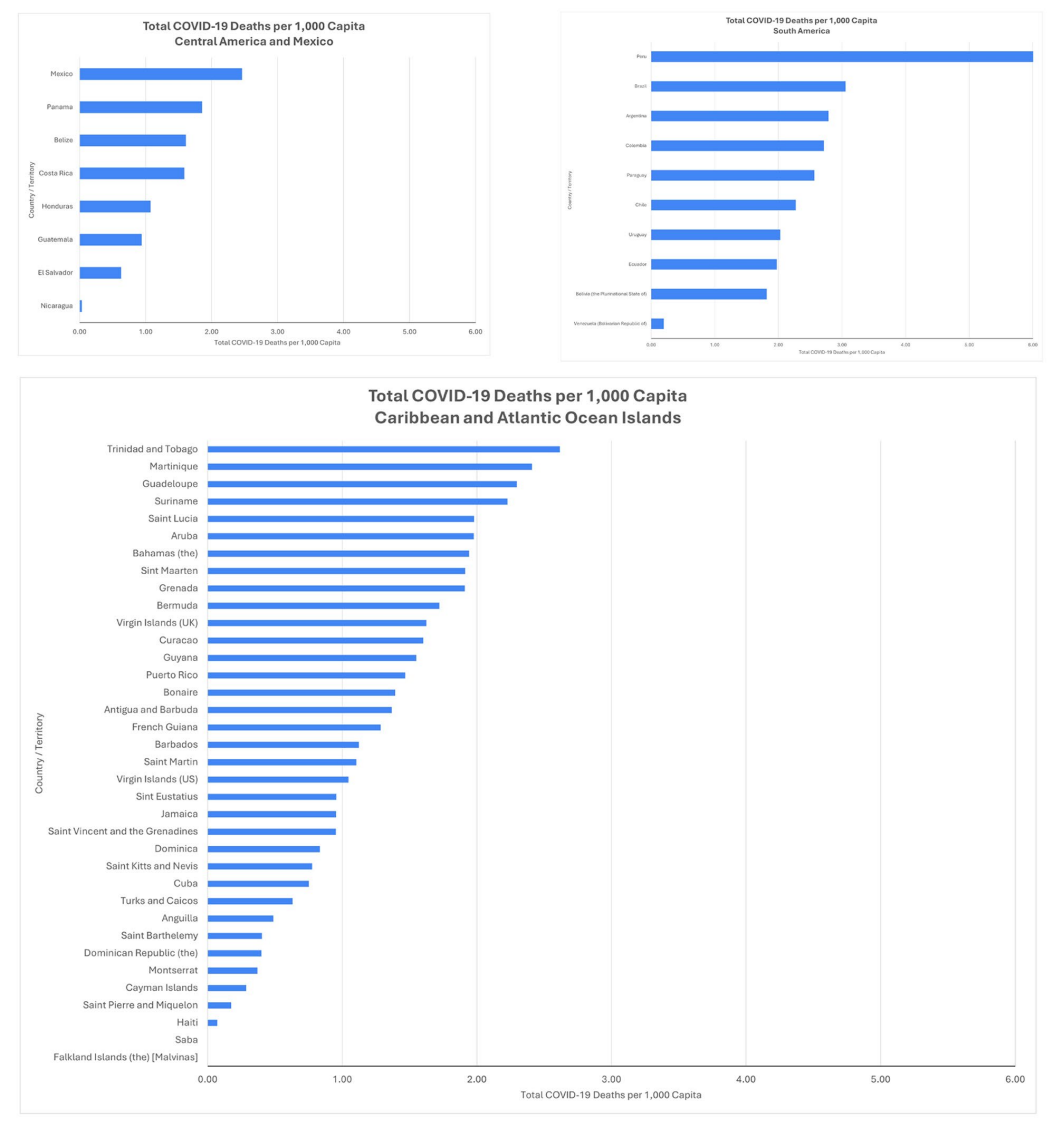
Total COVID-19 Deaths Per 1,000 Capita as of 3/12/22, by Sub-Region. Source: https://ais.paho.org/imm/IM_DosisAdmin-Vacunacion.asp; https://www.worldometers.info/; https://ais.paho.org/phip/viz/COVID19Table.asp

The COVID-19 pandemic landed in this landscape, precipitating an unprecedented public health crisis. Despite advances, several countries suffered outsized impacts as health systems buckled under unmanageable caseloads. Figure 1 captures the differential impact of COVID-19 in the first two years of the pandemic, within LAC sub-regions. Peru had the greatest number of deaths per capita, more than double the impact elsewhere in the region. Many Caribbean countries, including Trinidad and Tobago, Martinique and Guadeloupe, experienced death rates per capita comparable to much larger regional neighbors. Further, closing non-COVID related health services, such as sexual and reproductive health and non-communicable disease (NCD) care, will have ripple effects for years to come.

### System-wide responses

For many countries, COVID-19 revealed that health care and public health institutions were weaker than had been assumed and increasingly lacked legitimacy [5–7], with tensions between health system decentralization and politics playing a major role. The COVID-19 policy response in LAC reflected the nature of political regimes, governance arrangements, social protection programs, and feeble efforts to address the needs of the most vulnerable [5]. Neoliberal market policies, presence of authoritarianism, and segmented systems, particularly for health and welfare, influenced the state response to the pandemic. In many countries, early government containment actions focused on enforcing lockdowns—even through repressive and heavy-handed measures—rather than expanding contact-tracing and ensuring continuous non-COVID healthcare. Emergency measures passed to address the pandemic were prone to abuses of power by authorities. For example, in Guatemala, a few months into lockdown, over 24,000 people had been detained by police for curfew infringement [8].

Most governments provided some social protection or economic relief to their citizens, but these measures were time-limited and weak [5], especially where countries had limited welfare systems. These actions demonstrate the sustained impact of neoliberalism: limited support for the most marginalized plus privatization of risk. In El Salvador, the government provided those unable to earn an income with a single emergency cash transfer of 300USD, as well as a freeze on water, electricity, internet and rent payments, enabled by a loan from the International Monetary Fund. However, these limited economic relief measures were combined with strict and forceful virus containment measures [5]. Other countries implemented programs that used mechanisms such as bank cards and electricity bills that posed access barriers for the most vulnerable, despite earlier experiences with pro-poor measures [9, 10].

While economic support tended to be limited, there is also evidence that it contributed to compliance with pandemic mitigation measures such as movement restrictions and social distance policies [11]. Similarly, countries that had strong welfare systems before the pandemic began were more successful in implementing pandemic-related measures. For example, Argentina and Chile had stronger social protection programs and the public’s compliance with mobility restriction policies was higher than in Colombia, Brazil, and Mexico [11]. A link between compliance with lockdown measures and poverty was also identified. Work mobility after lockdown announcements decreased less in areas with higher poverty rates, suggesting that poor workers relying on informal work for basic needs could not risk staying home [12]. Concerningly, there was a correlation between less mobility reduction in impoverished areas and higher COVID-19 spread [12]. Analyses from early pandemic measures also suggest that countries with greater trust and communication from government (Argentina, Chile and Colombia) led to greater mobility reduction. Meanwhile elsewhere ineffective communications including presidential messages that contradicted health authorities and minimized the pandemic resulted in less compliance with public health measures [11].

Brazil’s COVID-19 response provides a striking example of how institutions in LAC are directly influenced by political dynamics. Brazil’s strong labor movement has contributed to its relatively strong welfare system, which has included one of the most widely recognized conditional cash transfer programs, the Bolsa Familia. When then President Jair Bolsonaro refused to admit the severity of COVID-19 and provide assistance to the poor, Congress passed measures to expand the Bolsa Familia program. Similarly, when the president opposed implementing national COVID restrictions such as quarantines, business closures, or social distancing measures, state governors and Congress had to manage the pandemic separately, with most states defying President Bolsonaro and implementing quarantines or social distancing guidelines. The misalignment between different levels and branches of the government led to “an uneven and uncoordinated response across the country” [5] as well as public conflict between authorities. For example, the Governor of Sao Paulo, João Doria, implemented strict COVID-19 measures, which led to public disagreement of governance: President Bolsonaro publicly spoke out against Governor Doria’s actions, while Doria accused the federal government of relegating its responsibilities [5].

A comparative study of Brazil, Mexico and the US highlights how political factors—more than epidemiological data or sociodemographic conditions—drove the variation of public health protection measures at state level: political affiliations, alliances between governors, and top-down pressures from central institutions meant more aligned and stringent protections [13]. Brazil presents a unique case because its subnational leaders have substantially more power than comparable authorities elsewhere, allowing them to defy presidential mandates; still, President Bolsonaro’s COVID-19 denialism, combined with disagreement from governors, led to a lack of systematic plans and response to contain COVID-19 [5].

At a global level, governance arrangements were negatively affected by politics. Despite being predicated on equity and cooperation, the COVAX Facility mechanism did not live up to its potential. Vaccine nationalism weakened the supply to COVAX while, simultaneously, vaccine diplomacy saw countries such as China, India and Russia pursue geopolitically motivated vaccine donations to strengthen political alliances and influence [14].

### Politics override evidence

COVID-19 put in stark relief how politics within the health system and the broader political environment affect the public’s health; nowhere is this more evident than in the battle between health evidence, politics, and the economy. LAC had in many ways been at the vanguard of pushing for evidence-informed decision-making, including legal mandates requiring evaluations of large-scale programs [15], experiments of policy-maker/researcher pairs for implementation research [16], strengthening structures using evidence in decisions [17], and organizational culture within Ministries of Health that value evidence in decision-making [18]. Yet, politics played an outsized role in decision-making around COVID-19. This phenomenon was partially driven by the rapidly evolving science and guidance, making it imperative that policymakers make crucial decisions with imperfect information. However, it enabled politicians to take advantage of uncertainty as a way to justify avoiding or delaying necessary public health measures.

In LAC, the pandemic also collided with the re-emergence of some political leaders who were motivated–at least rhetorically–by anti-elitism, combating entrenched political parties and corrupt leaders [19]. Pandemic conditions aligned well with the anti-intellectualist stance of some governments and created opportunities for political leaders to dismiss evidence as needed, such as Presidents Bolsonaro’s denial of the existence of the virus in Brazil [20]. In parallel, the public was also losing confidence and trust in official data with numerous reports of epidemiology surveillance institutions delaying or making mistakes in reporting of cases and deaths [6, 21, 22], reports of official data manipulation to present the pandemic as less severe [21, 23], and shutting down access to public information [24].

Elsewhere, evidence was used to justify exerting further control over government and society. Prevention measures for COVID-19 took on a security approach where the state apparatus was used to control the population, often with heavy-handed measures to limit people’s movement, imposing forced quarantine and isolation, suspending civil rights [5], and using state of emergency declarations to claim extra-constitutional power [19].

Many governments pushed to keep industry and the economy open in lieu of broad containment and prevention measures, jeopardizing the health and welfare of the most marginalized workers. For instance, in Guatemala, *maquilas* were classified as a priority by authorities and remained operational despite lockdowns in the rest of the economy [25], causing COVID-19 outbreaks among workers [26]. In Peru, national authorities exempted the mining industry from lockdowns [27], in spite of the rigid and coercive measures for all other industries and populations [28]. Even in Chile, early policy decisions were slower than elsewhere in the region and oscillated between keeping businesses open and various containment measures. The government’s goal of keeping the economy active faced considerable resistance from municipal authorities, workers, and medical experts; eventually, high COVID-19 caseloads forced a return to more broad-based restrictions [5].

### Corruption

In many cases, corruption worsened during the pandemic as existing regulations were relaxed or co-opted under the guise of crisis response, with considerable implications for health systems. Investigative journalists reported corruption in public procurement related to COVID-19 in at least ten countries in the region. The most common cause of corruption was ad hoc health regulations to benefit specific vendors of medicines and other essential supplies or building infrastructure for medical care (Uruguay [29], Guatemala [30–32], Honduras [33], Brazil [34]). In addition, several countries, including El Salvador and Mexico, suspended public access to information regulations and purposely avoided established transparency mechanisms for public procurement [35, 36].

Corruption was also exacerbated through authoritarian practices of governments, particularly the executive branch. For instance, in Guatemala and El Salvador authorities closed established spaces for governance and attacked other public institutions in charge of guaranteeing a system of checks and balances and independent monitoring of public actions [37, 38]. Journalists also reported that authorities actively obstructed access to information on beneficiaries of government emergency programs aimed at reducing the negative effects of the pandemic [39, 40].

Meanwhile, government officials were also found to establish privileges for themselves, families and friends to be first in line in receiving COVID-19 vaccines, therapeutics and specialized medical care [41, 42] or redirect public funds to dubious expenses, such as investments at private health facilities and catering services [43, 44]. In general, large infusions of funding to be spent quickly have been difficult to account for and track.

### Building back health systems

Uneven progress and persistent inequalities demonstrate how technical measures, including high quality health care and best practice public policies like UHC, cannot be successful “if bolted to a dysfunctional structure dominated by entrenched power of groups that resist reform” [45]. As governments failed to meet their social contract, especially for the needs of marginalized populations, the efforts needed to rebuild trust in health systems and broader institutions are substantial. Prioritizing the economy over public health–especially where critical industries rely on low-income workers–weakened community trust. COVID-19 social support measures highlighted the need to better integrate health systems and social protection policies, especially as it relates to informal sector workers.

Health systems governance and institutions need to meaningfully institutionalize representation from stakeholders from all walks of life so that in times of crisis, short-term decision-making is informed by diverse perspectives. Recommitting to transparency for data systems and evidence-informed decisions is also essential given the politicization of the pandemic response. Local governments, such as regional and municipal authorities, have an important role to play in a crisis response and recovery, both to respond to local needs but also to rebuild trust in public officials and institutions. The belated engagement of sub-national authorities in decision-making, especially in the early days of COVID-19, cannot be a hallmark of things to come. Reaffirming the distribution of responsibilities between central and local authorities will be critical to increase accountability for implementing public health measures between different levels of the health system as well as between the public and government. Relatedly, civil society organizations can play an integral role in responding to local needs, mediating the dialogue between citizens and the state, and leveraging data for accountability.

Likewise, transparent communication and exchange between authorities and the public is necessary. A lack of public trust in institutions is associated with limited state capacity in LAC, and transparent communication that contributes to trust between authorities, institutions and the public is crucial for successfully implementing policies, developing state capacity, and strengthening health systems [46]. It is unclear if pre-pandemic mechanisms for dialogue will ever be fully reinstated. Civil society efforts to build these were substantial already, and governments may be concerned that reopening dialogue spaces will once again increase scrutiny and demands for transparency. There is an important research agenda to pursue around transparent communication exchange that contributes to trust between authorities and the public.

### Implications for global health and health policy and systems research

What does this mean for global health and health policy and systems research (HPSR)? First, clearly new frameworks and metrics are needed to understand and strengthen health systems at national and global levels. The measures employed to define strong health systems failed - or at least the assumptions underlying those metrics did not withstand a true test. The poor predictive ability of the Global Health Security Index, which pre-COVID ranked the US and UK as having the highest capacity to respond to infectious disease threats, showed that pre-pandemic assessments were deeply naive to how health systems evidence and expertise are uniquely vulnerable to politics. As Dalglish critiqued in early 2020, COVID-19 undermined all the arguments of the supremacy of high-income country expertise [47].

Expanding our research repertory and practice can help. For example, research into the political economy dynamics that contributed to COVID-19 response breakdowns and successes are necessary. Likewise, applied research that contributes to building or revising frameworks can ground our understanding in reality. HPSR is critical for programs and policies, but current investment does not reflect this. Worse still, funding for HPSR is impacted by political shifts and the broader political environment, especially as funds are redirected towards COVID research and away from other areas [48].

Second, HPSR also needs to rethink its narratives about health systems. There is a deep tension about what health systems need to deliver now versus a vision of what health systems could be. Given its central role in health, systems discussions get lost in a sea of competing needs of individual programs areas (e.g., maternal and child health, NCDs, vaccination). One way to highlight the multiplicative effect of health systems investments is drawing attention to the excess mortality and increased disability-adjusted life years resulting from suspending and delaying non-COVID-19 services [49].

Typical discussions about health systems do not concretely convey to policymakers the critical investments that are needed. We echo others’ concerns about the risk of pandemic preparedness overtaking systems investment–as seen in West Africa after the 2014 Ebola crisis–and that “lessons learned” will focus on technical interventions and ignore the political nature of health [50]. HPSR needs a vision for health systems that account for post-pandemic realities, and we are already running behind. Understanding what policymakers need to know coupled with language for concrete actions and integrated activities should be a priority.

Lastly, the implications of COVID-19 for the politics of global health governance cannot be understated. Discourse and aspirations about parity between countries are only lip service if high-income countries always act out of self-interest, rather than solidarity. We face an existential question: what does it mean for the future of cooperation and the Sustainable Development Goals if wealthier countries pursued vaccine nationalism at the first opportunity? Even in the face of evidence that cross-border cooperation was better for international epidemiological control, self-interest won the day and continues still. Geopolitics, intellectual property rights, and country regulatory standards have run headlong into cooperation goals, and HPSR needs to understand these incentives better.

We need research and co-creation to develop new models for global health governance and cooperation, which requires understanding the breakdowns that COVID-19 precipitated as well as opportunities for innovation. New arrangements that support global governance and cooperation, and specifically address the incentives of self-serving politics and work to minimize them are crucial for successful reform [51]. While reforming global health governance is complicated and has been limited by a multiplicity of actors with competing interests, the deep costs of COVID-19 and fear of future pandemics deliver an unprecedented window of opportunity.

## Data Availability

No datasets were generated or analysed during the current study.
